# Residual antiseptic efficacy of octenidine dihydrochloride versus chlorhexidine gluconate in alcoholic solutions

**DOI:** 10.1186/2047-2994-4-S1-P33

**Published:** 2015-06-16

**Authors:** FHH Brill, N Radischat, P Goroncy-Bermes, J Siebert

**Affiliations:** 1 Dr. Brill + Partner GMBH Instiute for Hygiene and Microbiology, Hamburg, Germany; 2 Research & Development, Schülke & Mayr GmbH, Norderstedt, Germany

## Introduction

Skin antisepsis is an important measure to prevent postoperative infections. It is known that wound infections infringe the well-being of the patient and prolongs the rehabilitation significantly. At the same time additional costs will be caused in the health care system. Alcohol-based solutions containing the active ingredients chlorhexidine gluconate [CHX] or octenidine dihydrochloride [OCT] have a residual antimicrobial effect on skin. This may re-sult in a better preventative outcome than alcohol alone.

## Objectives

The aim of the presented study was to compare the immediate and long-term efficacy of CHX and OCT.

## Methods

We performed a study on the skin on the arm of 20 healthy volunteers in a cross-over design based on DGHM-standard method 13 and measured the colony forming units (cfu) of the resident skin flora after the application of the products according to Williamson and Kligman (1965). The cfu were determined directly after the application and after 24 h on 5 consecutive study days. The calculated log reduction factors were statistically evaluated with the student’s t-test.

## Results

Both solutions showed as expected a significant any quick reduction of the resisdent skin flora and a long-term effect over 24 h. For OCT a statistically significant superior long-term effect after four applications was determined (lg reduction: (2.21 vs. 1.37; p = 0.004).

## Conclusion

The presented data show that alcoholic solutions with octenidin dihydrochloride and chlorhexidine gluconate show a comparable efficacy on the resident skin flora. Alcohol-based preparations with the additional active octenidin dihydrochloride are a valid alternative for skin antisepics to the world-wide broadly used CHX-based products.

## Disclosure of interest

F. Brill Grant/Research support from: partially by Schülke & Mayr, Germany., N. Radischat Employee of: Schülke & Mayr, Germany., P. Goroncy-Bermes Employee of: Schülke & Mayr, Germany., J. Siebert Employee of: Schülke & Mayr, Germany.

